# SR9883 is a novel small-molecule enhancer of α4β2* nicotinic acetylcholine receptor signaling that decreases intravenous nicotine self-administration in rats

**DOI:** 10.3389/fnmol.2024.1459098

**Published:** 2024-09-05

**Authors:** Kevin M. Braunscheidel, George Voren, Christie D. Fowler, Qun Lu, Alexander Kuryatov, Michael D. Cameron, Ines Ibañez-Tallon, Jon M. Lindstrom, Theodore M. Kamenecka, Paul J. Kenny

**Affiliations:** ^1^Nash Family Department of Neuroscience, Icahn School of Medicine at Mount Sinai, New York, NY, United States; ^2^Friedman Brain Institute, Icahn School of Medicine at Mount Sinai, New York, NY, United States; ^3^Department of Neurobiology and Behavior, University of California, Irvine, Irvine, CA, United States; ^4^The Herbert Wertheim UF Scripps Institute for Biomedical Innovation and Technology, Jupiter, FL, United States; ^5^Department of Neuroscience, Perelman School of Medicine at the University of Pennsylvania, Philadelphia, PA, United States; ^6^The Laboratory of Molecular Biology, The Rockefeller University, New York, NY, United States

**Keywords:** nicotinic acetylcholine receptors, α4β2* nAChRs, NS9283, SR9883, nicotine addiction, smoking cessation, intracranial self-stimulation (ICSS), intravenous self-administration (IVSA)

## Abstract

**Background:**

Most smokers attempting to quit will quickly relapse to tobacco use even when treated with the most efficacious smoking cessation agents currently available. This highlights the need to develop effective new smoking cessation medications. Evidence suggests that positive allosteric modulators (PAM) and other enhancers of nicotinic acetylcholine receptor (nAChR) signaling could have therapeutic utility as smoking cessation agents.

**Methods:**

3-[3-(3-pyridyl)-1,2,4-oxadiazol-5-yl]benzonitrile (NS9283) was used as a starting point for medical chemistry efforts to develop novel small molecule enhancers of α4β2* nAChR stoichiometries containing a low-affinity agonist binding site at the interface of α4/α4 and α4/α5 subunits.

**Results:**

The NS9283 derivative SR9883 enhanced the effect of nicotine on α4β2* nAChR stoichiometries containing low-affinity agonist binding sites, with EC_50_ values from 0.2–0.4 μM. SR9883 had no effect on α3β2* or α3β4* nAChRs. SR9883 was bioavailable after intravenous (1 mg kg^−1^) and oral (10–20 mg kg^−1^) administration and penetrated into the brain. When administered alone, SR9883 (5–10 mg kg^−1^) had no effect on locomotor activity or intracranial self-stimulation (ICSS) thresholds in mice. When co-administered with nicotine, SR9883 enhanced locomotor suppression and elevations of ICSS thresholds induced by nicotine. SR9883 (5 and 10 mg kg^−1^) decreased responding for intravenous nicotine infusions (0.03 mg kg^−1^ per infusion) but had no effect on responding for food rewards in rats.

**Conclusions:**

These data suggest that SR9883 is useful for investigating behavioral processes regulated by certain α4β2* nAChR stoichiometries. SR9883 and related compounds with favorable drug-like physiochemical and pharmacological properties hold promise as novel treatments of tobacco use disorder.

## Introduction

Smoking-related illnesses such as chronic obstructive pulmonary disorder (COPD) and lung cancer are leading causes of premature death in the United States (Mathers and Loncar, 2006; Doll et al., 2004). Smokers who achieve long-term abstinence before the onset of tobacco-related illness can largely avoid this increased mortality risk (Peto et al., 2000; Doll et al., 1994). Nevertheless, the majority of smokers seeking to quit will relapse to tobacco use within 4 weeks of attempted abstinence (Benowitz, 2009). Currently available smoking cessation agents have modest clinical efficacy (Harmey et al., 2012; Lengel and Kenny, 2023). For example, approximately 23% of people treated with Chantix (varenicline) and 16% treated with Zyban (bupropion) remained abstinent after 1 year of a quit attempt, compared with approximately 9% of placebo-treated individuals (Knight et al., 2009). Pharmacotherapy is therefore an effective strategy to aid smoking cessation, but considerable risk of relapse remains even when using the most clinically efficacious smoking cessation agents currently available. This highlights the pressing need to develop new efficacious smoking cessation agents.

Nicotine is the major reinforcing component of tobacco responsible for the initiation and maintenance of cigarette smoking (Stolerman and Jarvis, 1995). Nicotine acts in the brain by stimulating nicotinic acetylcholine receptors (nAChRs) (Wills et al., 2022). Neuronal nAChRs are composed of five discrete membrane-spanning subunits (Wills et al., 2022; Lena and Changeux, 1998; Albuquerque et al., 1995). The neuronal α nAChR subunit exists in eight major isoforms (α2-α7 and α9-α10), with six expressed in the brain (α2-α7) (Wills et al., 2022; Elgoyhen et al., 2001; Elgoyhen et al., 1994; Le Novere et al., 2002). The α8 nAChR subunit is found in avian but not in mammalian tissues (Dani, 2015). The β nAChR subunit exists in three isoforms in the brain (β2-β4) (Wills et al., 2022; Elgoyhen et al., 2001; Elgoyhen et al., 1994; Le Novere et al., 2002). Neuronal nAChRs composed of α4 and β2 subunits (denoted as α4β2* nAChRs; asterisk indicates possible presence of other subunits) are the predominant subtype in mammalian brain (Deneris et al., 1991; Sargent, 1993; Flores et al., 1992). α4β2* nAChRs account for the majority of high-affinity nicotine binding sites and play a major role in regulating behavioral responses to nicotine (Wills et al., 2022; Tapper et al., 2004; Picciotto et al., 1998). Nicotine and other nAChR agonists bind at the interface between α and β subunits (orthosteric sites) to stabilize the open (active) conformation of the receptor complex (Lena and Changeux, 1993; Changeux et al., 1984; Changeux, 2018). Full and partial nAChR agonists differ in terms of their efficacy in stabilizing the active receptor conformation (Lena and Changeux, 1993; Changeux et al., 1984; Changeux, 2018), with full but not partial agonists achieving the same maximal efficacy induced by the cognate agonist acetylcholine. Nicotine and other nAChR agonists also facilitate the transition of the receptor complex from an active to desensitized (inactive) state (Lena and Changeux, 1993; Changeux et al., 1984; Changeux, 2018). The smoking cessation agent varenicline (a synthetic derivative of cytisine) is a partial agonist at α4β2* nAChRs (Coe et al., 2005; Lerman et al., 2007). The rationale for developing varenicline was two-fold: First, as a partial agonist varenicline should compete with nicotine for the orthosteric binding sites on α4β2* nAChRs and thereby attenuate the reinforcing properties of nicotine (Coe et al., 2005; Reperant et al., 2010). Second, low levels of α4β2* nAChR signaling induced by varenicline should partially substitute for nicotine during abstinence (Coe et al., 2005), thereby counteracting withdrawal-related responses that contribute to relapse (Coe et al., 2005; Kenny and Markou, 2001). These pharmacological features of varenicline are thought to underlie its therapeutic utility as a smoking cessation agent (Coe et al., 2005; Lerman et al., 2007). The clinical efficacy of varenicline suggests that other agents that enhance α4β2* nAChR signaling could serve as treatments for tobacco use disorder.

Neuronal α4β2* nAChRs can assume two discrete subunit stoichiometries: (α4)_2_(β2)_2_β2 or (α4)_2_(β2)_2_α4 nAChRs (Wills et al., 2022; Wang et al., 2015; Mazzaferro et al., 2011; Harpsoe et al., 2011). The (α4)_2_(β2)_2_β2 nAChR subtype contains two high-affinity acetylcholine/nicotine binding sites at the interface of α4/β2 subunits (Wang et al., 2015; Mazzaferro et al., 2011; Harpsoe et al., 2011). The (α4)_2_(β2)_2_α4 nAChR subtype also contains two high-affinity binding sites along with a third low-affinity site at the α4/α4 interface (Wang et al., 2015; Mazzaferro et al., 2011; Harpsoe et al., 2011). Likewise, α4β2* nAChRs containing an α5 accessory subunit assume a (α4)_2_(β2)_2_α5 stoichiometry with a low-affinity binding site at the α4/α5 interface (Wang and Lindstrom, 2018; Jain et al., 2016). NS9283 is a small molecular weight compound that was initially thought to bind to non-orthosteric sites on α4β2* nAChRs and exert a positive allosteric modulator (PAM) effect (Grupe et al., 2013; Timmermann et al., 2012; Zhu et al., 2011). Instead, NS9283 binds specifically to the low-affinity α4/α4 and α4/α5 interfaces but not the high-affinity α4/β2 interface contained in (α4)_2_(β2)_2_α4/α5 nAChR stoichiometries (Wang et al., 2015; Grupe et al., 2013; Timmermann et al., 2012; Olsen et al., 2014; Marotta et al., 2014; Appiani et al., 2024; Olsen et al., 2013). Alone, NS9283 is unable to activate α4β2* nAChRs (Wang et al., 2015; Jain et al., 2016), but it can enhance the activity of (α4)_2_(β2)_2_α4/α5 nAChR stoichiometries after their stimulation by acetylcholine, nicotine, and other agonists that bind to the high-affinity α4/β2 interface (Wang et al., 2015). This is reflected by a left-ward shift in the concentration-response functions of agonist-induced stimulation of (α4)_2_(β2)_2_α4/α5 nAChRs in the presence of NS9283, without any alteration in the absolute maximal efficacy of the response (Wang et al., 2015). The half-maximal effective concentration (EC_50_) of NS9283 on α4β2* nAChR signaling is reported to be approximately 1 μM (Wang et al., 2015; Grupe et al., 2013; Appiani et al., 2024; Jin et al., 2014). Notably, NS9283 has been shown to decrease intravenous nicotine self-administration behavior in rats (Maurer et al., 2017). This raises the possibility that NS9283 and other related compounds that act at the low-affinity binding sites contained in some α4β2* nAChR stoichiometries could represent a novel class of smoking cessation agents.

We used NS9283 as the starting point for medical chemistry efforts to identify novel higher potency enhancers of α4β2* nAChR signaling (Jin et al., 2014). The 3-pyridyl ring, isoxazole core, and 3-cyanophenyl ring in NS9283 were systematically substituted while seeking to maintain pharmacological activity and brain penetration (Jin et al., 2014). These efforts yielded 3-(5-(pyridin-3-yl)-2H-tetrazol-2-yl)benzonitrile (SR9883), which potently enhanced the activity of (α4)_2_(β2)_2_α5 nAChRs (Jin et al., 2014). Here, we report that SR9883 enhances the function of α4β2* nAChRs containing α4 or α5 accessory subunits but not α3β2* or α3β4* nAChRs. SR9883 was bioavailable after systemic administration, and readily penetrated into the brain. SR9883 enhanced the locomotor-suppressing and intracranial self-stimulation (ICSS) threshold-elevating actions of nicotine, consistent with increased behavioral responses to nicotine. Finally, SR9883 decreased nicotine self-administration behavior in rats.

## Materials and methods

### Animals

Male mice with null mutation in the α5 nAChR subunit gene (*Chrna5^−/−^* mice) and wild-type littermates (*Chrna5^+/+^* mice) were bred for at >10 generations on a C57BL/6 J background and were maintained by mating heterozygous pairs. Mice were genotyped according to published protocols (Fowler et al., 2011). Male mice were housed in groups of 2–3 per cage (littermates; mixed genotypes) until surgical implantation of IV catheters or intracranial self-stimulation (ICSS) stimulating electrodes, when all mice were housed 1 per cage. Mice used for experiments were between 8 and 12 weeks of age. Male Wistar rats (Charles River Laboratories, Raleigh, NC) were housed in groups of 1–2 per cage throughout. Rats weighed 300 g at the start of experiments. All animals were maintained in an environmentally controlled vivarium on a 12 h: 12 h reversed light/dark cycle. Food and water were provided *ad libitum* to mice and rats until behavioral training commenced. All procedures were conducted in strict accordance with the NIH Guide for the Care and Use of Laboratory Animals and were approved by the Institutional Animal Care and Use Committees of Scripps Florida and the Icahn School of Medicine at Mount Sinai.

### Drugs

(−)-Nicotine hydrogen tartrate salt (Sigma) was dissolved in 0.9% sterile saline. Doses of nicotine throughout refer to the free-base form. Nicotine was dissolved in 0.9% sterile saline and was administered acutely by subcutaneous (SC) injection or chronically by subcutaneously implanted osmotic minipumps (see below). SR9883 trifluoroacetate salt was synthesized in-house (T.M.K.) and was dissolved in DMSO (10%) for cell assays, DMSO: Tween-80: Water (10:10:80 ratio; v:v:v) vehicle for pharmacokinetic studies, or DMSO:Cremophor:Water vehicle (10:10:80 ratio; v:v:v) for behavioral studies. SR9883 was administered acutely by intravenous (IV), oral (PO), or intraperitoneal (IP) injection as indicated. Systemically administered drugs were delivered in a volume of 1 and 10 mL kg^−1^ of body weight in rats and mice, respectively.

### Cell culture and FLEXstation

Human embryonic kidney (HEK) cell lines stably expressing human (α4)_2_(β2)_2_β2/α4 (α4β2*) nAChRs, (α4)_2_(β2)_2_α5 nAChRs, (α4)_2_(β2)_2_β3 nAChRs, (α3)_2_(β4)_2_α3/β4 (α3β4*) nAChRs, (α3)_2_(β2)_2_α3/β2 (α3β2*) nAChRs, (α3)_2_(β4)_2_α5 nAChRs, or (α3)_2_(β2)_2_α5 nAChRs were maintained as described previously (Wang et al., 2015; Kuryatov et al., 2005; Caligiuri et al., 2022). The FLEXstation bench top scanning fluorometer from Molecular Devices was used to assay nAChR function in these cells, according to published protocols (Wang et al., 2015; Kuryatov et al., 2005). The membrane potential kit (Molecular Devices) was used according to the manufacturer’s protocols. Serial dilutions of SR9883 were manually added to the assay plate 15 min prior to recording. EC_20-30_ concentrations of nicotine (ranging from 0.1–3 μM) that elicited 20–30% of the maximal response evoked by acetylcholine were used to assess the facilitatory effects of SR9883 on nAChR signaling. Compound dilutions were prepared in V-shaped 96-well plates (Fisher Scientific Co.) and added to cell culture wells at a rate of 20 μL s^−1^ during recording. Cells were incubated at 29°C for 6–20 h prior to assays to upregulate nAChRs (Wang et al., 2015; Kuryatov et al., 2005). Data points were averaged from 3 to 4 responses from separate wells. Potency and efficacy were calculated using the Hill equation as described previously (Wang et al., 2015).

### Pharmacokinetics

Mice prepared with intravenous catheters and treated with SR9883 via oral (10 mg kg^−1^) or intravenous (1 mg kg^−1^) routes of administration. Blood samples were collected up to 240 min after treatment into lithium heparin coated tubes (Ram Scientific) and stored on wet ice. Blood samples were later centrifuged for 3 min at 5,000 RPM to separate plasma from red blood cells. Plasma was collected into a fresh tube and stored at −80°C. A separate group of mice was treated with SR9883 (20 mg kg^−1^ PO) and killed by cervical dislocation 120 min later. Their brains were collected and stored at −80°C. SR9883 levels were quantified in plasma and brain by mass spectrometry using an ABSciex 5,500 instrument and using multiple reaction monitoring as previously described (Radnai et al., 2021). Brain samples were homogenized in saline and then immediately treated with twice the amount (v:v) of acetonitrile to extract the compound. Plasma samples were directly treated with acetonitrile. Samples were filtered through a 0.2 μm filter plate prior to injection onto the LC–MS/MS as previously described (Radnai et al., 2021).

### Locomotor activity

Locomotor activity was assessed in an open-field apparatus (40 cm × 40 cm; Omnitech Electronics Inc.) under low illumination (~30 radiometric lux). Mice were habituated to the apparatus for 60 min on the day before treatments commenced. On test days, mice received a total of two systemic injections. First, mice were injected intraperitoneally with SR9883 (0, 5, 10 mg kg^−1^) and returned to their home cages. Then ~5 min later they were injected subcutaneously with saline or nicotine (0.25 mg kg^−1^) and returned to their home cage for ~25 min session. Mice were then placed in the center of the apparatus and the total distance they traveled was recorded for 60 min (Fusion software, Omnitech Electronics Inc.).

### ICSS electrode implantation and testing procedure

Mice were anesthetized with a mixture of isoflurane (1–3%) and oxygen vapor then positioned in a stereotaxic frame in the flat-skull position (Kopf Instruments) (Fowler et al., 2013; Johnson et al., 2008). A stainless-steel bipolar electrode (Plastics One) was implanted into the lateral hypothalamus according to the following stereotaxic coordinates: Anterior/Posterior: −0.5 mm from bregma; Medial/Lateral: ±1.3 mm from midline; Dorsal/Ventral: −5.0 mm from brain surface (Fowler et al., 2013; Johnson et al., 2008; Paxinos and Franklin, 2001). Mice were permitted 72 h to recover from surgery prior to training in the ICSS procedure. Mice were permitted to respond for intracranial electrical stimulation according to a modification of the discrete-trial current-threshold procedure developed by Kornetsky and colleagues (Gill et al., 2004), as previously described (Fowler et al., 2013; Johnson et al., 2008). The electrical reinforcer had a train duration of 500 ms and consisted of 0.1 ms square wave pulses that were delivered at a frequency of 25 to 100 Hz. The frequency of the stimulation was selected for each mouse so that current-intensity thresholds were within 25 to 400 μA, permitting threshold elevations and reductions to be reliably detected. This frequency for each mouse was held constant throughout the experiment. A one-quarter turn of the wheel manipulandum within 7.5 s of the delivery of the non-contingent electrical stimulation resulted in the delivery of a stimulus identical in all parameters. After a variable intertrial interval (7.5–12.5 s, average of 10 s), another trial was initiated with the delivery of a non-contingent electrical stimulus. Failure to respond to the non-contingent stimulus within 7.5 s resulted in the onset of the inter-trial interval. Responding during the inter-trial interval reset the interval and delayed the onset of the next trial. Current levels were varied in alternating descending and ascending series. A set of five trials was presented for each current intensity. Current intensities were altered in 5 μA steps. In each testing session, four alternating descending and ascending series were presented. The threshold for each series was defined as the midpoint between three consecutive current intensities that yielded “positive scores” (animals responded for at least three of the five trials) and two consecutive current intensities that yielded “negative scores” (animals did not respond for three or more of the five trials). The overall threshold of the session was defined as the mean of the thresholds for the four individual series. Each testing session was approximately 45 min in duration. The time between the onset of the non-contingent stimulus and a positive response was recorded as the response latency. The response latency for each test session was defined as the mean response latency of all trials during which a positive response occurred. After establishment of stable ICSS reward thresholds (defined as ≤15% variation in thresholds over a 3-day period), mice were tested in the ICSS procedure once daily.

### Osmotic minipump implantation

Once the ICSS-responding mice established stable reward thresholds (<20% variation in thresholds across three consecutive days requiring 7–10 daily sessions) they were anesthetized with isoflurane and subcutaneously implanted with osmotic minipumps (Alzet; model 2004; 28-day pumps) delivering saline or nicotine. The concentration of the nicotine was adjusted according to animal body weight to deliver 24 mg kg^−1^ per day (free-base). The surgical wound was sutured following implantation of the minipump, and mice were administered the analgesic Metacam (0.2 mg/kg^−1^, meloxicam, Boehringer Ingelheim). ICSS threshold assessments recommenced 24 h after implantation of osmotic minipumps and continued during daily sessions. Effects of intraperitoneal injections of SR9883 on ICSS thresholds were assessed >7 days after minipump implantation.

### Food responding and intravenous nicotine self-administration procedures

Operant chambers (Med Associates, East Fairfield, VT, United States) were used to assess responding by rats for food pellets and intravenous nicotine infusions. The chambers were equipped with two response levers (designated active and inactive) with a cue light located above each lever, a food pellet dispenser located between the levers, and a computer-controlled injection pump for the scheduled delivery of nicotine infusions through an intravenous catheter. All rats were food-restricted to maintain their body weight at approximately 85% that of free-feeding weights. They were then trained to press an active lever for 45 mg food pellets on a fixed ratio 5 time-out 20 s (FR5TO20) schedule of reinforcement. An inactive lever was also extended in the operant chamber throughout the session. Responding the inactive lever was recorded but had no scheduled consequence. Rats responded for food rewards until a reliable responding was achieved, defined as >90 pellets earned per 60 min session across three consecutive sessions. Rats that were used in nicotine self-administration experiments were prepared with intravenous catheters. Rats were anesthetized using 1–3% isoflurane inhalation in oxygen and catheters were implanted into the left jugular vein. The catheter was passed subcutaneously to a polyethylene assembly mounted on the animals’ back. Rats were then permitted to respond under the same FR5TO20 schedule of reinforcement for food or nicotine infusions (0.03 mg kg^−1^ per infusion delivered over 1 s) during 60 min daily sessions. Delivery of each food or nicotine reinforcer initiated a 20 s time-out period, signaled by a light cue located above the active lever. During the time period, responding on the active lever had no scheduled consequence. After each nicotine session, catheters were flushed with heparinized saline (30 U per mL) and checked for leaks or blockages. Rats received intraperitoneal injections of SR9883 or vehicle (0.3 mL per 300 g weight) 30 min prior to the food or nicotine sessions.

### Statistical analyses

Concentration-response curves of SR9883 actions in nAChR-expressing HEK cells and oocytes were fitted to the Hill equation using a nonlinear least squares curve fit method and efficacy and potency values were calculated as previously described (Wang et al., 2015). Plasma and brain levels of SR9983 after oral administration were compared using a two-tailed paired t test. The effects of SR9883 on locomotor activity alone or in combination with nicotine injection were assessed by two-way repeated-measures analysis of variance (ANOVA). For the ICSS threshold experiment, percentages of baseline reward threshold scores were calculated by expressing the absolute threshold scores as a percentage of the scores obtained during baseline. The baseline values were the mean thresholds on the three sessions prior to drug injections. Response latency data were analyzed in the same manner as the threshold data. Effects of SR9883 on ICSS thresholds in the mice implanted with saline or nicotine minipumps were assessed by two-way repeated-measures ANOVA. Effects of SR9883 on responding for food pellets in rats was analyzed by a two-tailed paired t test. Effects of SR9883 on responding for nicotine infusions in rats were analyzed by one-factor repeated-measures ANOVA. Significant main or interaction effects in ANOVAs were followed by Bonferroni or Newman–Keuls post-hoc tests as appropriate. All statistical analyses were performed using GraphPad Prism software with α set to 0.05.

## Results

### SR9883 enhances α4β2* nAChR signaling

**Figure 1:**
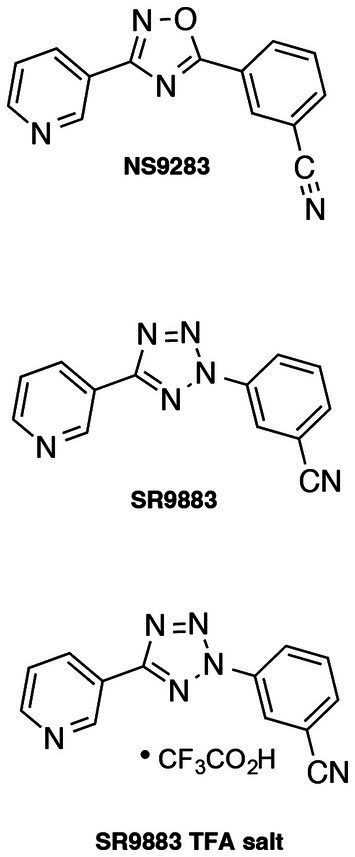
Relevant chemical structures. Structures of NS9283 and SR9883, including the trifluoroacetate (TFA) salt of SR9883.

Previously, we reported that NS9283 enhanced the effects of acetylcholine and nicotine on (α4)_2_(β2)_2_α4, (α4)_2_(β2)_2_α5, and (α4)_2_(β2)_2_β3 stoichiometries of nAChRs but not the (α4)_2_(β2)_2_β2 nAChR stoichiometry (Wang et al., 2015; Jain et al., 2016). This is consistent with a stimulatory effect of NS9283 on the low-affinity α4/α4, α4/α5, and α4/β3 interfaces that are absent in (α4)_2_(β2)_2_β2 nAChRs (Wang et al., 2015). The EC_50_ of NS9283 on α4β2* nAChRs is ~1 μM (Grupe et al., 2013; Appiani et al., 2024; Jin et al., 2014). NS9283 was used as the starting point for medical chemistry efforts to identify novel high-potency enhancers of α4β2* nAChR signaling with favorable physiochemical properties (Jin et al., 2014; Figure 1). Synthesis of SR9883 was carried out as previously described (Jin et al., 2014). The 3-pyridyl ring, isoxazole core, and 3-cyanophenyl ring in NS9283 were systematically substituted with a key focus on maintaining pharmacological activity and brain penetration (Jin et al., 2014). These efforts yielded SR9883 [(5-(pyridin-3-yl)-2H-tetrazol-2-yl)benzonitrile] (Jin et al., 2014).

We assessed the effects of a broad range of SR9883 concentrations (0.0005–10 μM) on HEK cells stably expressing (α4)_2_(β2)_2_β2/α4 (α4β2*), (α4)_2_(β2)_2_α5, or (α4)_2_(β2)_2_β3 nAChRs. The cells were treated with a fixed concentration of nicotine that elicited 20–30% of the maximal response evoked by acetylcholine (EC_20-30_ concentration; 0.2–3 μM range). SR9883 markedly enhanced the response of these nAChR stoichiometries to nicotine (Table 1). The EC_50_ concentrations of SR9883 on α4β2*, (α4)_2_(β2)_2_α5, and (α4)_2_(β2)_2_β3 nAChRs were ~ 0.4, ~0.3, and ~ 0.2 μM, respectively (Table 1), with ~3 μM SR9883 inducing maximal enhancement of nAChR signaling. The same concentrations of SR9883 had no effect on the response to EC_20-30_ concentrations of nicotine of HEK cells stably expressing (α3)_2_(β4)_2_α3/β4 (α3β4*) or (α3)_2_(β2)_2_α3/β2 (α3β2*) nAChRs (Table 1). Likewise, SR9883 had no effect on the response to EC_20-30_ concentrations of nicotine of HEKs expressing (α3)_2_(β4)_2_α5 or (α3)_2_(β2)_2_α5 nAChRs (Table 1).

**Table 1:** Effect of a broad range of SR9883 concentrations (0.0005–10 μM) on the response to an EC_20-30_ concentration of nicotine in HEK293 cells expressing α4β2*, α3β4*, or α3β4* nAChR stoichiometries.

	EC_50_ (μM)	n_Hill_	Efficacy (%)
(α4)_2_(β2)_2_α4/β2 [α4β2*]	0.3811 ± 0.0916	1.1015 ± 0.2269	108.92 ± 7.65
(α4)_2_(β2)_2_α5	0.3212 ± 0.0982	0.8620 ± 0.1564	146.18 ± 10.93
(α4)_2_(β2)_2_β3	0.2240 ± 0.1490	0.8567 ± 0.3792	96.44 ± 15.93
(α3)_2_(β4)_2_α3/β4 [α3β4*]	No enhancement		
(α3)_2_(β4)_2_α5	No enhancement		
(α3)_2_(β2)_2_α3/β2	No enhancement		
(α3)_2_(β2)_2_α5	No enhancement		

Shown are the EC_50_ values (±SEM) and Hill coefficients (n_Hill_) (±SEM) of SR9883. Shown also is the change in the % maximal efficacy of response to nicotine in the presence of SR9883 (±SEM). Efficacy was calculated as the percentage response evoked by an EC_20–30_ concentration of nicotine in the presence of SR9883 relative to the response evoked by nicotine alone.

Next, we used a fixed concentration of SR9883 (10 μM) and characterized the response of α4β2* and (α4)_2_(β2)_2_α5 nAChRs to a broad range of nicotine concentrations (0.001–50 μM). SR9883 enhanced the response of these nAChRs to nicotine, reflected by a reduction in the EC_50_ concentrations of nicotine (Table 2). SR9883 had no effect on maximum response to nicotine in these cells (i.e., efficacy of nicotine was unchanged) (Table 2). These data suggest that SR9883 enhances the function of stoichiometries of α4β2 nAChRs that contain low-affinity binding sites without alternating the activity of any stoichiometry of α3* nAChRs.

**Table 2:** Effect of SR9883 (10 μM) on the response of α4β2* and (α4)_2_(β2)_2_α5 nAChR stoichiometries to a wide range of nicotine concentrations (0.001–50 μM).

Stoichiometry	Treatment	EC_50_ (μM)	n_Hill_	Efficacy (%)
α4β2	−SR9883	0.2589 ± 0.3515	0.8145 ± 0.0740	96.89 ± 2.82
	+SR9883	0.0121 ± 0.0011	1.0749 ± 0.0928	87.20 ± 1.68
(α4)_2_(β2)_2_α5	−SR9883	0.3460 ± 0.0622	0.7395 ± 0.0767	100.49 ± 3.89
	+SR9883	0.0194 ± 0.0019	1.2934 ± 0.1439	92.69 ± 2.19

Shown are the EC_50_ (±SEM) values and Hill coefficients (n_Hill_) (±SEM) of nicotine. Shown also is the change in the % maximal efficacy of response to nicotine in the presence of SR9883 (±SEM). Efficacy was calculated as the percentage response evoked by an EC_20–30_ concentration of nicotine in the presence of SR9883 relative to the response evoked by nicotine alone.

### SR9883 is bioavailable and brain-penetrant after systemic administration

**Figure 2:**
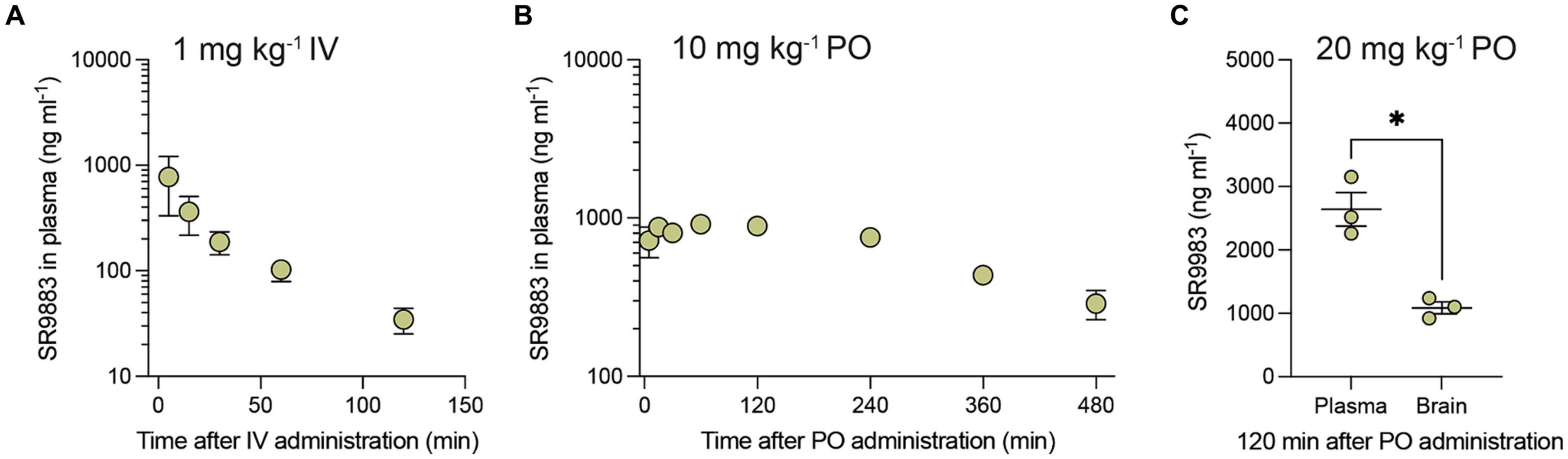
Pharmacokinetic and brain penetration profile of SR9883 in mice. **(A)** Time-course of changes in the concentrations of SR9883 detected in plasma (ng mL^−1^) of male C57BL/6J mice (*n* = 3) following IV (1 mg kg^−1^) administration. **(B)** Time-course of changes in concentrations of SR9883 detected in plasma (ng mL^−1^) in C57BL/6J mice (*n* = 3) following oral (10 mg kg^−1^) administration. **(C)** Concentrations (ng mL^−1^) of SR9883 detected in plasma and brain of C57BL/6J mice (*n* = 3) following oral (20 mg kg^−1^) administration. All data are expressed as mean (±S.E.M.) SR9883 concentrations (ng ml^−1^). *p < 0.05, unpaired two-tailed t test. See text for conversion of ng mL^−1^ to μM.

Bioavailability of SR9883 was assessed in mice by quantifying plasma and brain concentrations after systemic administration. SR9883 (1 mg kg^−1^) had a maximum plasma concentration (C_max_) of ~775 ng ml^−1^ (~3 μM) and a plasma half-life (T_½_) of approximately 53 min after intravenous (IV) administration (Figure 2A; Table 3). SR9883 (10 mg kg^−1^) had a C_max_ of 1,015 ng ml^−1^ (~4 μM) a and a T_½_ of ~64 min after oral (PO) administration (Figure 2B; Table 3). To assess brain penetration, mice were treated with SR9883 (20 mg kg^−1^ PO) and their plasma and brains collected 120 min later. Plasma SR9883 levels were ~ 2,600 ng ml^−1^ (~11 μM) and brain levels were ~ 1,100 ng ml^−1^ (~4.5 μM) (Figure 2C; Table 3). These data confirm that SR9883 enters the plasma after IV or PO administration and readily penetrates into the brain.

**Table 3:** Pharmacokinetic profile of SR9883 after intravenous (IV) or oral (*per os*; PO) administration to male C57BL/6J mice (*n* = 3 in each case).

	T_1/2_ (hr)	T_max_ (hr)	C_max_ (ng mL^−1^)	C_max_ (μM)	AUC_last_ (μM.hr)	Cl_obs (mL min-1 kg^−1^)	Vss_obs (L kg^−1^)
IV (1 mg kg^−1^)	0.88 ± 0.3515	0.08 ± 0.0	773.67 ± 442.38	3.11 ± 1.78	1.77 ± 0.62	45.69 ± 12.83	2.72 ± 0.89
PO (10 mg kg^−1^)	3.73 ± 0.61	1.08 ± 0.51	1015.00 ± 60.62	4.07 ± 0.24	21.12 ± 0.54	24.59 ± 2.42	ND

Values are reported as mean (S.E.M.). T_1/2_, compound half-life; T_max_, Time of maximum observed concentration; C_max_, maximum observed concentration; AUC_last_, area under the curve up to the last quantifiable time-point; Cl_obs, clearance based on observed C_last_; Vss_obs, volume of distribution at steady state based last observed concentration; ND, not determined.

### SR9883 enhances the locomotor-suppressant effects of nicotine

**Figure 3:**
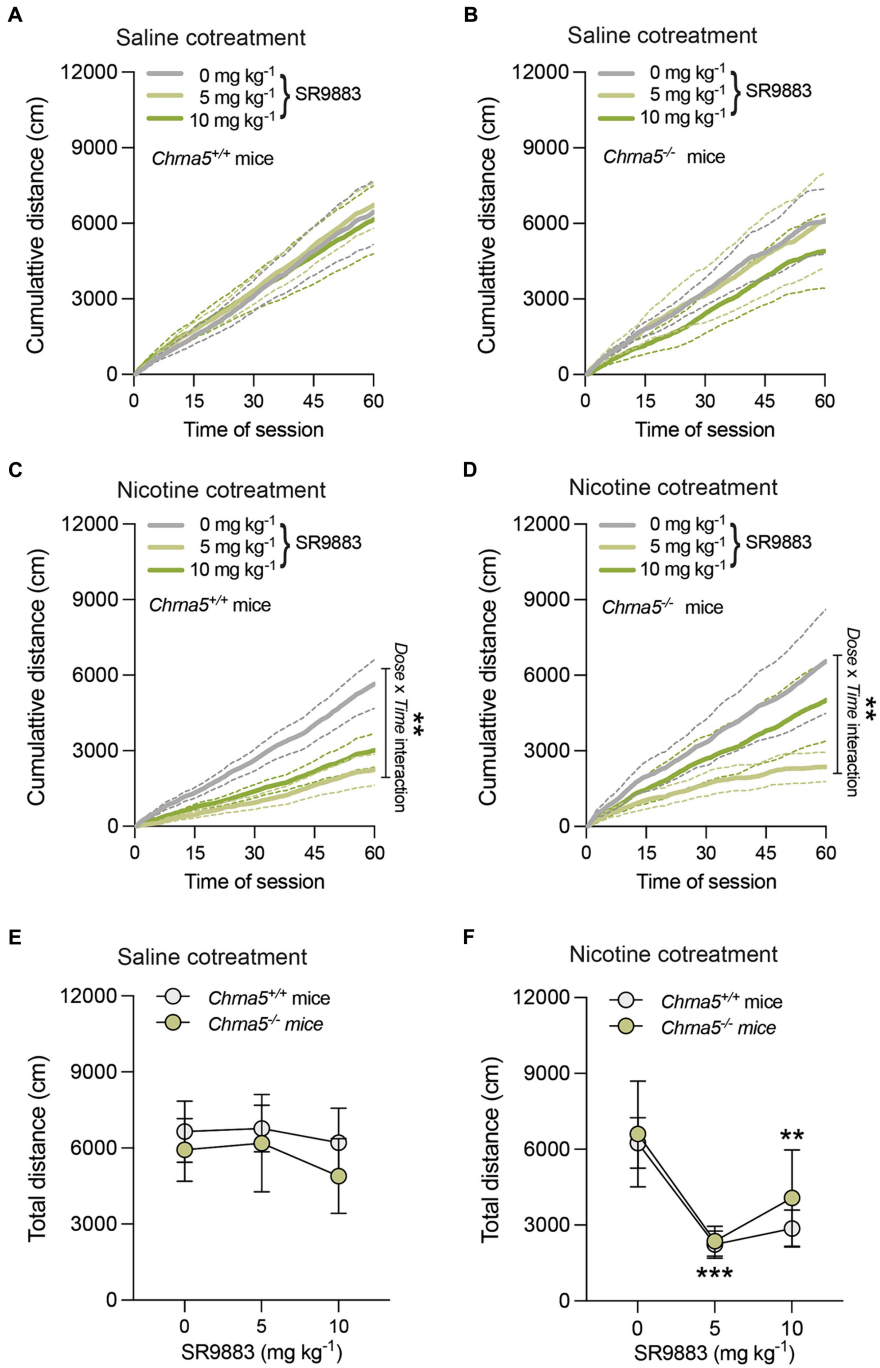
SR9883 enhances the locomotor-suppressing effects of nicotine in mice. The effects of SR9883 on locomotor activity were assessed in male *Chrna5^+/+^* and *Chrna5^−/−^* mice during 60 min sessions. *Chrna5^+/+^* mice (*n* = 9) **(A)** and *Chrna5^−/−^* mice (*n* = 5) **(B)** were pretreated with SR9883 (0, 5, or 10 mg kg^−1^) then injected with saline before testing. Data are expressed as mean (±S.E.M.) cumulative distance traveled (cm) during the 60 min session. *Chrna5^+/+^* mice (*n* = 9) **(C)** and *Chrna5^−/−^* mice (*n* = 5) **(D)** were pretreated with SR9883 (0, 5, or 10 mg kg^−1^) then injected with nicotine (0.25 mg kg^−1^) before testing. Data are expressed as mean (±S.E.M.) cumulative distance traveled (cm) during the 60 min session. **p < 0.01; significant interaction effect between *Dose* and *Time* in two-way repeated-measures ANOVA. **(E)** Locomotor activity in saline-treated *Chrna5^+/+^* and *Chrna5^−/−^* mice after SR9883 injection (0, 5, or 10 mg kg^−1^). Data are expressed as mean (±S.E.M.) total distance traveled (cm). **(F)** Locomotor activity in nicotine-treated *Chrna5^+/+^* and *Chrna5^−/−^* mice after SR9883 injection (0, 5, or 10 mg kg^−1^). Data are expressed as mean (±S.E.M.) total distance traveled (cm). **p < 0.01, ***p < 0.001 compared with vehicle treatment; post-hoc comparisons after significant main effect of SR9883 treatment in two-way repeated-measures ANOVA.

The effects of SR9883 alone or in combination with nicotine on locomotor activity were assessed in mice. As SR9883 enhances the stimulatory effects of nicotine on (α4)_2_(β2)_2_α4 and (α4)_2_(β2)_2_α5 nAChR stoichiometries, we used α5 nAChR subunit gene knockout mice (*Chrna5^−/−^* mice) and their wild-type littermates (*Chrna5^+/+^* mice) for this experiment. In this manner, the importance of the α5 nAChR accessory subunit in regulating the effects of SR9883 could be assessed. Cumulative distance traveled during the 60 min session was unchanged by SR9883 (5 or 10 mg kg^−1^) treatment in saline-injected *Chrna5^+/+^* mice (Figure 3A). This was reflected by the absence of main or interaction effects SR9883 dose in a two-way repeated measures ANOVA [*Dose*: *F*_(1.527, 12.21)_ = 0.069, *p* = 0.98897; *Time*: *F*_(120, 660.0)_ = 37.69, *p* = 0.0001; *Dose* x *Time*: *F*_(1.790, 14.32)_ = 0.3122, *p* = 0.7134]. Similarly, cumulative distance traveled was unchanged by SR9883 in saline-injected *Chrna5^−/−^* mice [*Dose*: *F*_(1.049, 4.194)_ = 2.304, *p* = 0.2015; *Time*: *F*_(120, 480.0)_ = 12.52, *p* = 0.0001; *Dose* x *Time*: *F*_(1.578, 6.313)_ = 1.033, *p* = 0.3893] (Figure 3B). Cumulative distance traveled was decreased by SR9883 in nicotine (0.25 mg kg^−1^ SC)-injected *Chrna5^+/+^* mice (Figure 3C). This was reflected by a statistically significant interaction effect between *Dose* and *Time* in a two-way repeated measures ANOVA [*Dose*: *F*_(1.873, 14.99)_ = 10.87, *p* = 0.0014; *Time*: *F*_(120, 960.0)_ = 32.49, *p* = 0.0001; *Dose* x *Time*: *F*_(1.988, 15.90)_ = 7.659, **p = 0.0047]. Cumulative distance traveled tended to be decreased by SR9883 in nicotine-injected *Chrna5^−/−^* mice (Figure 3D), but the interaction effect between *Dose* and *Time* failed to achieve statistical significance [*Dose*: *F*_(1.198, 4.794)_ = 3.716, *p* = 0.1127; *Time*: F_(120, 480.0)_ = 11.55, *p* = 0.0001; *Dose* x *Time*: *F*_(1.307, 5.229)_ = 4.033, *p* = 0.0946]. Next, we directly compared the effects of SR9883 on locomotor activity in *Chrna5^+/+^* vs. *Chrna5^−/−^* mice. We observed no difference in total distance traveled between saline-injected *Chrna5^+/+^* and *Chrna5^−/−^* mice after SR9883 treatment (Figure 3E). This was reflected by the absence of main or interaction effects in a two-way repeated measures ANOVA [*Dose*: *F*_(1.574, 18.88)_ = 0.7943, *p* = 0.4385; *Genotype*: *F*_(1, 12)_ = 0.2474, *p* = 0.6279; *Dose* x *Genotype*: *F*_(2, 24)_ = 0.1255, *p* = 0.8826]. Total distance traveled was decreased by a similar magnitude in nicotine-injected *Chrna5^+/+^* and *Chrna5^−/−^* mice after SR9883 treatment (Figure 3F) [*Dose*: *F*_(2, 24)_ = 11.87, *p* = 0.0003; *Genotype*: *F*_(1, 12)_ = 0.2062, *p* = 0.6578; *Dose* x *Genotype*: *F*_(2, 24)_ = 0.2116, *p* = 0.8108]. Bonferroni *post-hoc* tests showed that locomotor activity was reduced in nicotine-injected *Chrna5^+/+^* and *Chrna5^−/−^* mice treated with 5 and 10 mg kg^−1^ doses of SR9883 (*p* = 0.0002 and *p* = 0.0049, respectively) compared to vehicle treatment (Figure 3F). As SR9883 enhanced behavioral responses to nicotine similarly *Chrna5^+/+^* and *Chrna5^−/−^* mice, this indicates that α5 nAChR subunits are not required for the *in vivo* actions of SR9883. This also suggests that low-affinity α4/α4 and/or α4/β3 interfaces remain present in *Chrna5^−/−^* mice.

### SR9883 enhances the ICSS threshold-elevating effects of nicotine

**Figure 4:**
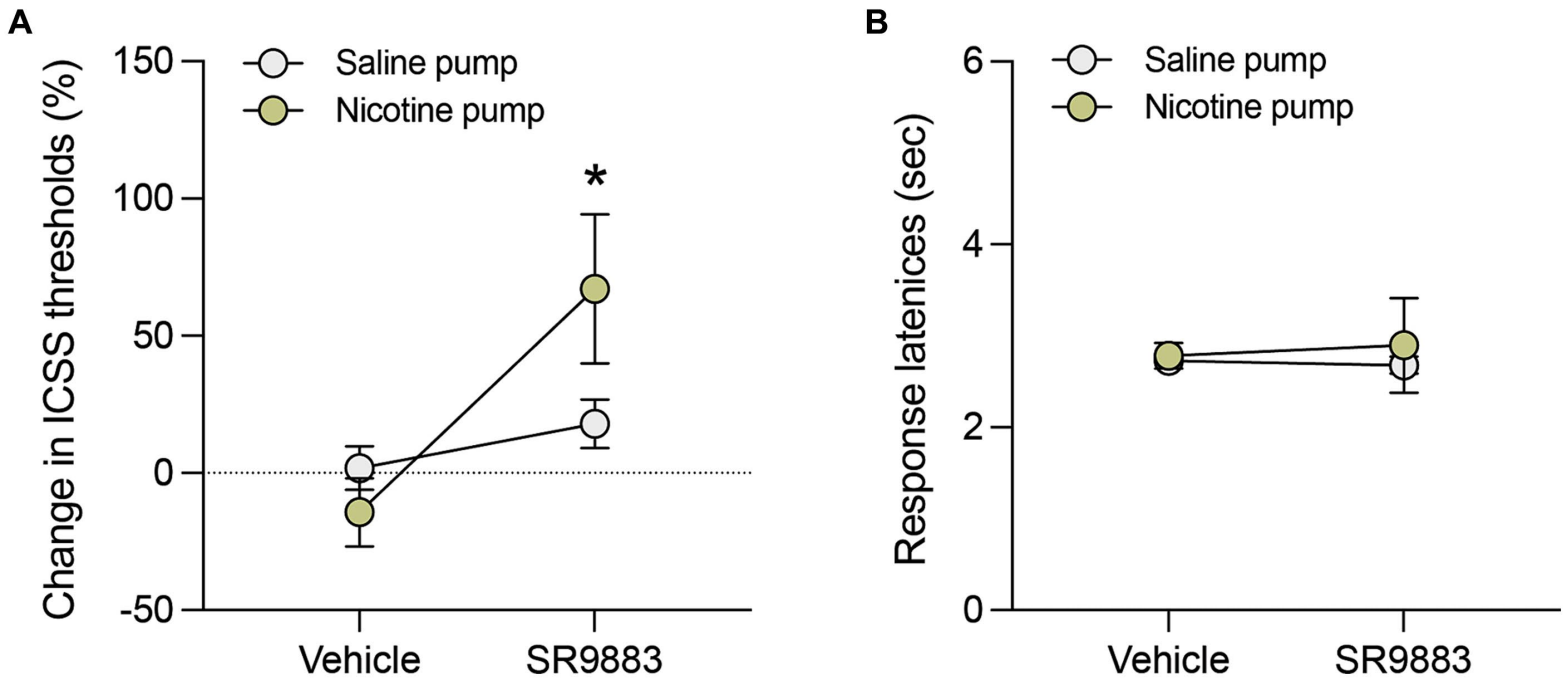
SR9883 enhances the reward-inhibiting effects of nicotine in mice. The effects of SR9887 on ICSS thresholds were tested in male C57BL/6J mice implanted with osmotic minipumps delivering saline (*n* = 3) or nicotine (*n* = 3; 24 mg kg^−1^ per day, free-base). **(A)** Mice were treated with vehicle or SR9883 (10 mg kg^−1^). Data are expressed as mean (±S.E.M.) percentage change from baseline ICSS thresholds. *p < 0.05 compared with vehicle treatment; post-hoc comparisons after significant main effect of SR9883 treatment in two-way repeated-measures ANOVA. **(B)** SR9883 had no effect on ICSS response latencies in mice with saline or nicotine-delivering osmotic minipumps. Data are expressed as mean (±S.E.M.) response latencies (sec).

Next, the effects of SR9883 alone or in combination with nicotine on ICSS thresholds were assessed. SR9883 (10 mg kg^−1^ IP) had no effect on ICSS thresholds in mice prepared with saline-delivering osmotic minipumps (Figure 4A). SR9883 (10 mg kg^−1^ IP) elevated ICSS thresholds in mice prepared with nicotine-delivering osmotic minipumps (Figure 4A). This was reflected by a main effect of SR9883 treatment in a two-way repeated measures ANOVA [*SR9883*: *F*_(1, 4)_ = 12.54, *p* = 0.0240; *Minipump*: *F*_(1, 4)_ = 0.8363, *p* = 0.4122; *SR9883* x *Minipump F*_(1, 4)_ = 5.640, *p* = 0.0764]. Bonferroni post-hoc tests showed that ICSS thresholds were elevated by SR9883 treatment relative to vehicle treatment in the mice with nicotine-delivering minipumps (*p* = 0.0278 and *p* = 0.9120, respectively) (Figure 4A). SR9883 had no effect on ICSS response latencies in mice with minipumps delivering either saline or nicotine (Figure 4B). This was reflected by the absence of main or interaction effects in a two-way repeated measures ANOVA [*SR9883*: *F*_(1, 4)_ = 0.02329, *p* = 0.8861; *Minipump*: *F*_(1, 4)_ = 0.1678, *p* = 0.7031; *SR9883* x *Minipump F*_(1, 4)_ = 0.1509, *p* = 0.7175]. These data show that SR9883 enhances the ICSS threshold-elevating response to nicotine, which is usually observed in mice and rats only when relatedly high doses of nicotine are administered (Fowler et al., 2011; Fowler et al., 2013).

### SR9883 decreases nicotine self-administration

**Figure 5:**
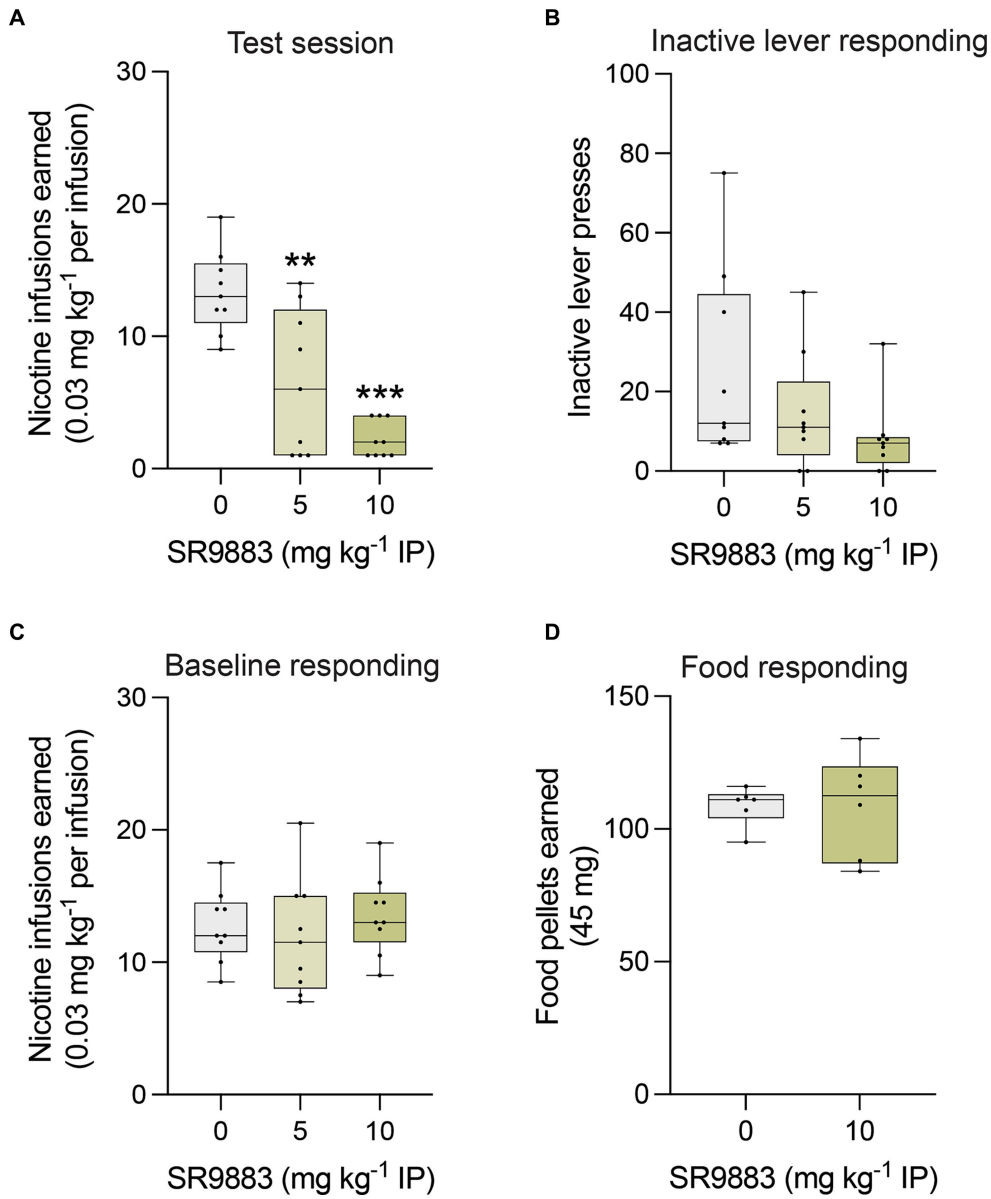
SR9883 decreases nicotine intake in rats. The effects of systemically administered SR9883 on responding for nicotine infusions (0.03 mg kg^−1^ per infusion) or food rewards were tested under FR5TO20 sec schedules of reinforcement. **(A)** Mean (±S.E.M.) number of nicotine reinforcers earned after treatment with SR9883 by male Wistar rats (*n* = 9). **p < 0.01 and ***p < 0.0001 compared with vehicle treatment; post-hoc test after a significant main effect in one-way repeated-measures ANOVA. **(B)** Mean (±S.E.M.) number of responses on the inactive lever after SR9883 treatment. **(C)** Mean (±S.E.M.) baseline number of nicotine reinforcers earned during the two daily sessions that proceeded each treatment with each dose of SR9883. **(D)** Mean (±S.E.M.) number of food pellets earned after treatment with SR9883 by male Wistar rats (*n* = 6).

We next assessed the effects of SR9883 on operant responding for nicotine infusions (0.03 mg kg^−1^ per infusion) under a FR5TO20 second schedule of reinforcement. SR9883 (5 and 10 mg kg^−1^ IP) dose-dependently decreased nicotine self-administration (Figure 5A), as reflected by a main effect of SR9883 treatment in a one-way repeated measures ANOVA [*F*_(1.490, 11.92)_ = 25.29, *p* < 0.0001]. Bonferroni post-hoc tests showed numbers of nicotine infusions earned by rats were decreased by 5 and 10 mg kg^−1^ SR9883 treatment relative to vehicle treatment (**p = 0.0021 and ***p < 0.0001, respectively) (Figure 5A). Responding on the inactive lever also tended to be decreased by SR9883 (Figure 5B), but this effect failed to achieve statistical significance [*F*_(1.435, 11.48)_ = 2.594, *p* = 0.1274]. Nicotine responding rapidly returned to baseline levels after SR9883 treatment and there were no residual deficits in responding across daily sessions (Figure 5C). This was reflected by the fact that mean numbers of nicotine infusions earned during the two daily self-administration session prior to each SR9883 treatment session remained unchanged across the course of the experiment (Figure 5C) [*F*_(1.872, 14.98)_ = 0.6410, *p* = 0.5307]. Finally, to determine whether SR9883 decreased nicotine intake by non-specifically disrupting operant performance, we assessed the effects of SR9883 on responding for food pellets in food-restricted rats tested under the same schedule of reinforcement. SR9883 (10 mg kg^−1^ IP) had no effect on food responding (Figure 5D), reflected by the absence of an effect in a two-tailed paired t test (*p* = 0.9868).

## Discussion

SR9883 is a potent and selective enhancer of α4β2* nAChRs that contain low-affinity agonist binding sites at the α4/α4 interface in the (α4)_2_(β2)_2_α4 nAChR stoichiometry, which are absent from the (α4)_2_(β2)_2_ β2 nAChR stoichiometry. SR9883 also enhances the function of α4* nAChRs that contain α5 or β3 accessory subunits, which contain low-affinity agonist binding sites at α4/α5 and α4/β3 interfaces, respectively. SR9883 has no effect on α3β2* or α3β4*nAChRs. SR9883 was bioavailable after IV and oral administration with a half-life of 50–60 min. SR9883 readily crossed the blood–brain barrier after oral administration to accumulate in the brain. Similar to NS9283 from which SR9883 is derived (Timmermann et al., 2012; Jin et al., 2014), SR9883 enhanced behavioral responses to nicotine that are known to be regulated by α4β2* nAChRs. This was reflected by enhanced sensitivity to nicotine-induced suppression of locomotor activity and nicotine-induced elevations of ICSS reward thresholds. Finally, SR9883 decreased intravenous nicotine self-administration at doses that had no effect on operant responding for food rewards.

NS9283 is reported to enhance agonist-induced α4β2* nAChR signaling with an EC_50_ value of ~1 μM (Grupe et al., 2013; Appiani et al., 2024; Jin et al., 2014). Consistent with these previous reports, we found that NS9283 enhanced nicotine-induced stimulation of α4β2* nAChR signaling with a similar EC_50_ value (Jin et al., 2014). SR9883 was identified during a medicinal chemistry campaign to optimize the drug-like physiochemical and pharmacological properties of NS9283 (Jin et al., 2014). All portions of NS9283 were modified and the effects on pharmacological activity at α4β2* nAChRs were assessed (Jin et al., 2014). Structure–activity relationships (SAR) were stringent, with most modifications to NS9283 resulting in the loss of pharmacological activity at α4β2* nAChRs (Jin et al., 2014). The 3-pyridyl ring was absolutely required for activity and tolerated no modifications (Jin et al., 2014), while substitutions of the 3-cyanophenyl ring yielded analogs exhibiting pharmacological activity similar to NS9283 (Jin et al., 2014). The N-linked tetrazole found in SR9883 was the only one of a dozen core modifications that maintained the pharmacological properties of NS9283 at α4β2* nAChR (Jin et al., 2014). The stringent SAR observed in this series likely reflects the challenge of targeting pharmacological agents to the low-affinity agonist binding site contained in certain stoichiometries of α4β2* nAChRs. SR9883 enhanced the stimulatory effects of nicotine on α4β2*, (α4)_2_(β2)_2_α5, and (α4)_2_(β2)_2_β3 nAChRs with EC_50_ values ranging from 0.2–0.4 μM. HEK cells stably expressing α4β2* nAChRs contain a mixture of two receptor stoichiometries: (α4)_2_(β2)_2_β2 and (α4)_2_(β2)_2_α4 nAChRs (Wang et al., 2015). In oocytes expressing pure α4β2* nAChRs stoichiometries, SR9983 enhanced nAChR-mediated currents in oocytes expressing (α4)_2_(β2)_2_α4 nAChRs but not (α4)_2_(β2)_2_β2 nAChRs (unpublished observations). SR9883 was detected at high concentrations in the plasma of mice after intravenous or oral administration. SR9883 was also detected in the brains of mice at relatively high concentrations after oral administration, with brain levels of SR9883 expected to enhance α4β2* nAChR signaling *in vivo*. Thus, SR9883 potently enhances the stimulatory effect of nicotine and acetylcholine on α4β2* nAChR stoichiometries that contain low-affinity binding sites at α4/α4, α4/α5, and α4/β3 subunit interfaces similar to the effects of NS9283 (Wang et al., 2015; Jin et al., 2014). SR9883 also penetrates into the brain after systemic administration. Based on these observations, we investigated whether SR9883 modifies behavioral responses to nicotine.

Doses of nicotine known to have aversive behavioral properties in rats and mice suppress locomotor activity, an action thought to reflect nicotine-induced malaise (Clarke and Kumar, 1983; Antolin-Fontes et al., 2020; Salas et al., 2003; Risinger and Oakes, 1995). Aversive reactions to nicotine are thought to promote nicotine avoidance behaviors (Fowler and Kenny, 2014). α3β4* nAChRs are known to play important roles in regulating aversive reactions to nicotine that suppress locomotor activity and promote nicotine avoidance behaviors (Salas et al., 2004; Kedmi et al., 2004; Elayouby et al., 2021). α5* nAChRs also contribute to aversion-related behavioral responses to nicotine (Fowler et al., 2011; Salas et al., 2004; Kedmi et al., 2004; Morton et al., 2018; Grieder et al., 2017; Xanthos et al., 2015). As SR9883 enhanced the stimulatory effect of nicotine on (α4)_2_(β2)_2_α5 nAChRs, we investigated the effects of SR9883 on locomotor activity in *Chrna5^+/+^* and *Chrna5^−/−^* (α5 nAChR subunit knockout) mice. Locomotor activity was unchanged in *Chrna5^+/+^* and *Chrna5^−/−^* mice after treatment with SR9883 alone (relative to vehicle treatment). Similarly, locomotor activity was unchanged in *Chrna5^+/+^* and *Chrna5^−/−^* mice after treatment with relatively low dose of nicotine (0.25 mg kg^−1^) that is known to have minimal aversive-related effects in mice (Fowler et al., 2013). However, locomotor activity was suppressed when SR9883 and nicotine were co-administered, with the magnitude of this effect similar in *Chrna5^+/+^* and *Chrna5^−/−^* mice. This suggest that SR9883 enhances the stimulatory effect of nicotine on α4β2* nAChRs to precipitate aversion-like suppression of locomotor activity. Further, these data suggest that α5 nAChR subunits are not required for SR9883 to enhance behavioral responses to nicotine and that SR9883 likely acts on α4/α4 and/or α4/β3 interfaces that are still present in *Chrna5^−/−^* mice.

In the ICSS procedure, rats and mice work vigorously to obtain rewarding electrical self-stimulation via an intracranial stimulating electrode, with the minimal stimulation intensity that maintains self-stimulation behavior termed the reward threshold (Kornetsky et al., 1979; Markou and Koob, 1992; Kenny, 2007). Relatively low doses of systemically administered nicotine (≤0.25 mg/kg^−1^) lower ICSS thresholds (Fowler et al., 2013; Kenny and Markou, 2006; Kenny et al., 2009). This action is thought to reflect an enhancement of brain reward activity by nicotine, which contributes to the positive motivational properties of the drug that support self-administration behavior (Kenny, 2007; Kenny and Markou, 2006). Conversely, higher doses of nicotine (>0.5 mg/kg) that precipitate aversion-related behaviors are known to elevate ICSS thresholds (Fowler et al., 2013; Schaefer and Michael, 1986). Rats and mice appear to titrate their nicotine self-administration behavior to maximize the stimulatory effects of nicotine on brain reward function while avoiding the reward-inhibiting actions of the drug at higher doses (Kenny and Markou, 2006; Kenny et al., 2009). We found that SR9883 had no effect on ICSS thresholds in mice prepared with subcutaneously implanted osmotic minipumps delivering saline. This suggests that SR9883 has no intrinsic effects on brain reward circuits. However, SR9883 precipitated a robust elevation of ICSS thresholds in mice implanted with minipumps delivering nicotine. This suggests that SR9883 enhanced the actions of nicotine in the brain to precipitate the same aversion-related elevations of ICSS thresholds that are usually only observed when animals are treated with high (aversive) nicotine doses (Fowler et al., 2011; Fowler et al., 2013). These data provide further support for enhanced stimulatory actions of nicotine on α4β2* nAChRs in animals treated with SR9883.

NS9283 was shown to decrease IV nicotine self-administration in rats (Maurer et al., 2017). Similarly, we found that SR9883 dose-dependently decreased nicotine self-administration in rats responding for nicotine infusions under a FR5TO20 sec schedule of reinforcement. SR9883 had no effect on operant performance in rats responding for food pellets under the same schedule of reinforcement. This suggests that decreased nicotine self-administration behavior in SR9883-treated rats was not secondary to non-selective deficits in behavioral performance. The inhibitory effects of NS9283 and SR9883 on nicotine self-administration in rats can be explained by at least two non-mutually exclusive mechanisms. First, these compounds could increase the stimulatory effect of nicotine on brain reinforcement circuits, resulting in lower quantities of self-administered nicotine being required to achieve the desired pharmacological actions of the drug (Maurer et al., 2017). Second, these compounds could increase the stimulatory effects of nicotine on brain satiety/aversion circuits (Fowler et al., 2011; Elayouby et al., 2021; Duncan et al., 2019; Tuesta et al., 2017), resulting in diminished reinforcing properties of nicotine and greater avoidance of the drug (Fowler et al., 2011; Caligiuri et al., 2022; Fowler et al., 2013; Fowler and Kenny, 2014; Elayouby et al., 2021; Duncan et al., 2019; Tuesta et al., 2017). Nicotine-induced suppression of locomotor activity (Clarke and Kumar, 1983; Antolin-Fontes et al., 2020; Salas et al., 2003) and elevations of ICSS thresholds (Fowler et al., 2011; Fowler et al., 2013) are thought to reflect the aversive properties of the drug that promote nicotine-avoidance and decrease consumption of the drug by rodents and humans (Wills et al., 2022; Fowler and Kenny, 2014). As described above, we found that SR9883 enhanced the sensitivity of mice to the locomotor-suppressing actions of nicotine. Similarly, SR9883 enhanced the sensitivity of mice to the ICSS threshold-elevating actions of nicotine. These findings suggest that SR9883 enhances the stimulatory effects of nicotine on α4β2* nAChRs located in brain satiety/aversion circuits and thereby decreases the motivational properties of the drug (Fowler et al., 2011; Caligiuri et al., 2022; Fowler et al., 2013; Fowler and Kenny, 2014; Elayouby et al., 2021; Duncan et al., 2019; Tuesta et al., 2017). If SR9883 and related compounds were to exert the same effect in human smokers, it might be expected that their tobacco consumption would be decreased while their ability to achieve abstinence would be increased. It should be noted that only male mice and rats were used in the present studies. Hence, it is possible that SR8883 and related compounds may have different effects on nicotine-related behaviors in female subjects relative to those reported here in males.

Overall, our data suggest that SR9883 is an agonist at the low-affinity binding site located at the interface of α4/α4 subunits contained in (α4)_2_(β2)_2_α4 but not the (α4)_2_(β2)_2_β2 stoichiometries of α4β2* nAChRs (Wang et al., 2015; Jain et al., 2016). SR9883 is also an agonist at the low-affinity binding site at the interface of α4/α5 and α4/β3 subunits in α4β2* nAChRs that contain α5 or β3 accessory subunits. SR9883 was bioavailable after systemic administration and readily penetrated into the brain. Furthermore, SR9883 markedly increased behavioral responses to nicotine, as reflected by enhanced sensitivity to the locomotor-suppressing and ICSS threshold-elevating actions of nicotine in mice. Moreover, SR9883 decreased nicotine self-administration in rats without precipitating deficits in operant performance or other non-specific actions. Hence, SR9883 is a pharmacological tool useful for studying α4β2* nAChR stoichiometries containing low-affinity binding sites and may serve as a substrate for future medicinal chemistry efforts to development novel treatments for tobacco use disorder.

## Data Availability

The datasets presented in this study can be found in online repositories. The names of the repository/repositories and accession number(s) can be found in the article/supplementary material.
